# Supplementary material for: A missense variant in SLC39A8 is associated with severe idiopathic scoliosis
Source: Nat Commun. 2018 Oct 9;9:4171. doi: 10.1038/s41467-018-06705-0 (PMC6177404; doi:10.1038/s41467-018-06705-0)
Supplement: Supplementary file 1 — Supplementary Information [file 41467_2018_6705_MOESM1_ESM.docx]

**SUPPLEMENTARY INFORMATION**

**TABLE OF CONTENTS**

| **Supplementary Figure 1** | **Gene diagram of *SLC39A8* with position of GWAS hits annotated** | **Page 2** |
| --- | --- | --- |
| **Supplementary Figure 2** | **Additional Images of abnormal slc39a8 KO zebrafish after alizarin red staining (9 months old)** | **Page 3** |
| **Supplementary Figure 3** | **Images of abnormal *slc39a8* KO zebrafish after alizarin red staining (12 weeks)** | **Page 4** |
| **Supplementary Figure 4** | **Expression of *slc39a8* mRNA revealed by whole-mount *in situ* hybridization** | **Page 5** |
| **Supplementary Figure 5** | **Calcein Staining of slc39a8 KO zebrafish and WT controls** | **Page 6** |
| **Supplementary Figure 6** | **Principal Components Analysis of Exome sequenced AIS cases, Controls and HapMap reference populations** | **Page 7** |
| **Supplementary Figure 7** | **Site Frequency Spectrum of SNPs in the discovery set** | **Page 8** |
| **Supplementary Table 1** | **Patient sample sources for discovery and replication cohorts and genotype/sequencing platforms used** | **Page 9** |
| **Supplementary Table 2** | **Zebrafish *in situ* probe sequence** | **Page 10** |


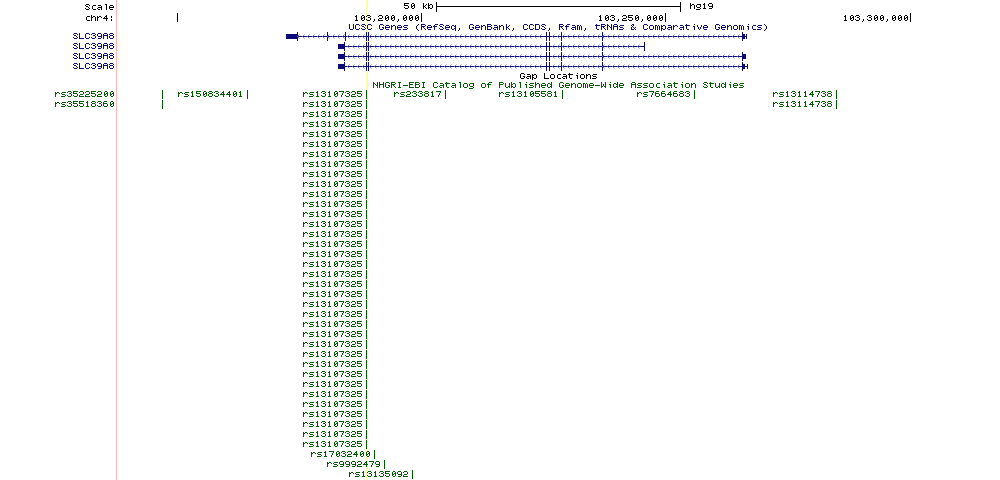


**Supplementary Figure 1. Gene diagram of SLC39A8 with position of GWAS hits annotated.** View of the SLC39A8 locus within the UCSC Genome browser with known genome-wide significant associations annotated with their rsIDs in green. Rs13107325 is listed once for each cited genome-wide significant association. SNPs are listed once for each independent genome-wide association publication in which they are listed.


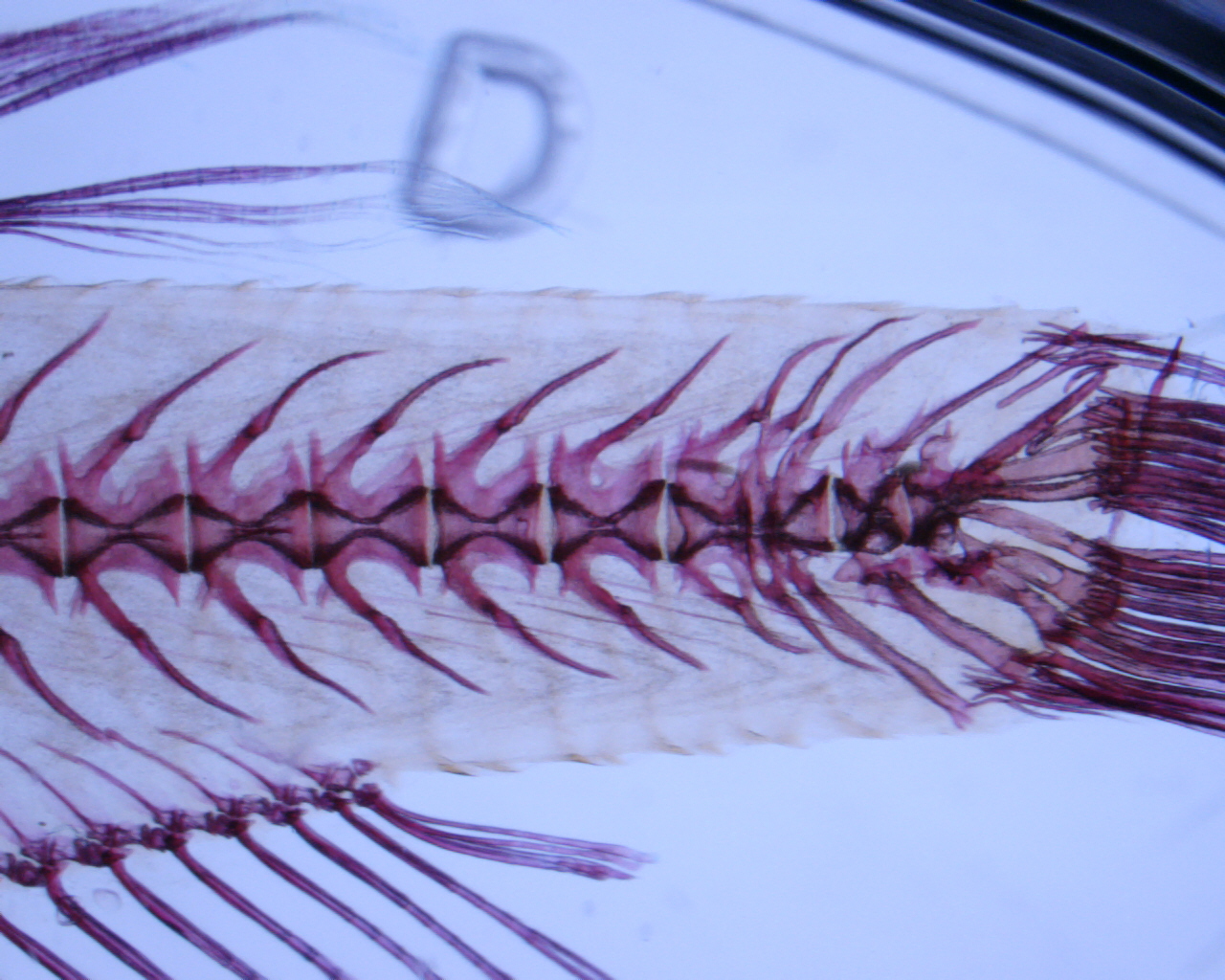


**Supplementary Figure 2. Additional Images of abnormal slc39a8 KO zebrafish after alizarin red staining.** Various skeletal abnormalities were observed among slc39a8 KO zebrafish including fused vertebrae and abnormal bone growth along the spine. Scale bar 1mm.

**
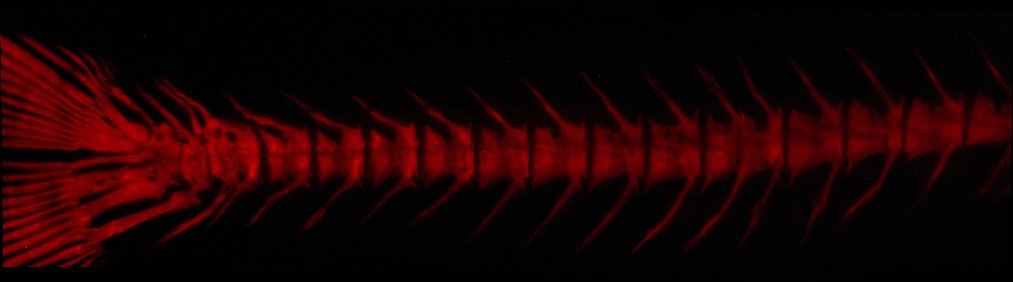
A**


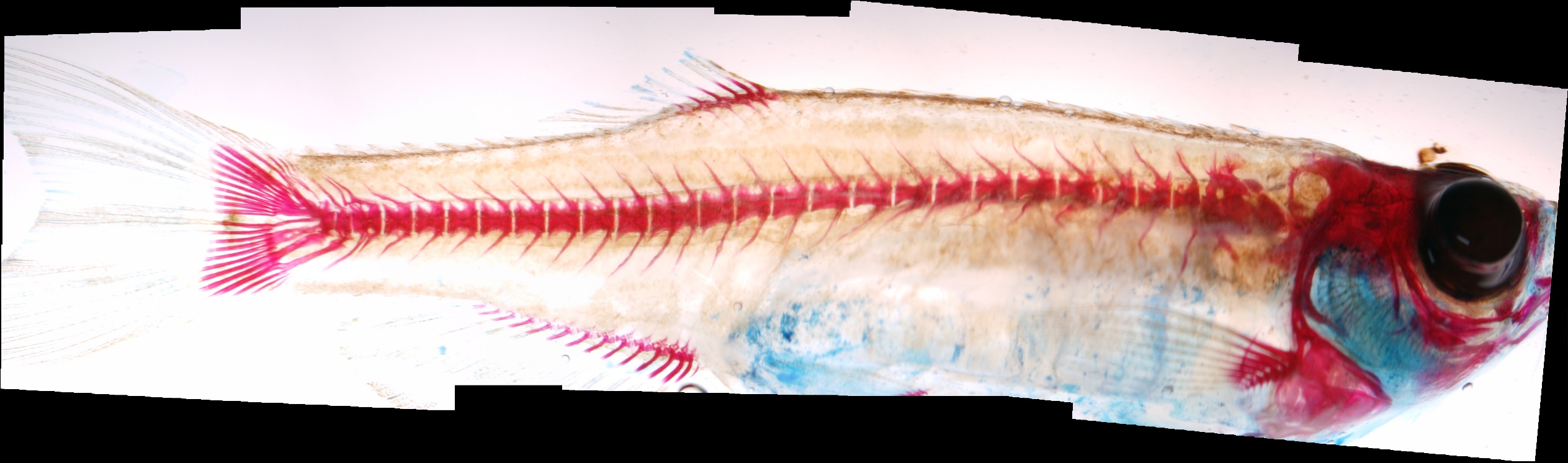


**B**


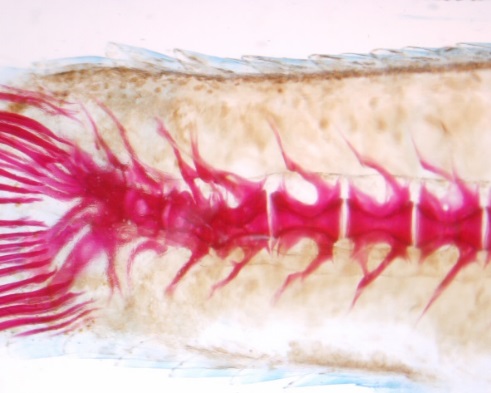

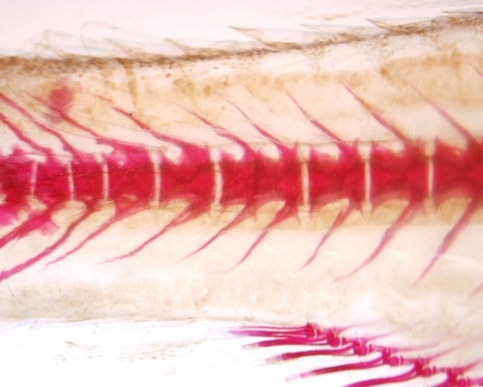

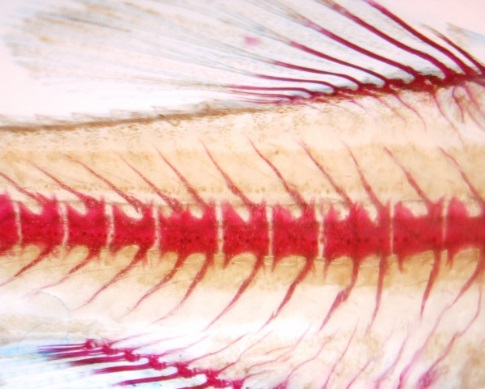

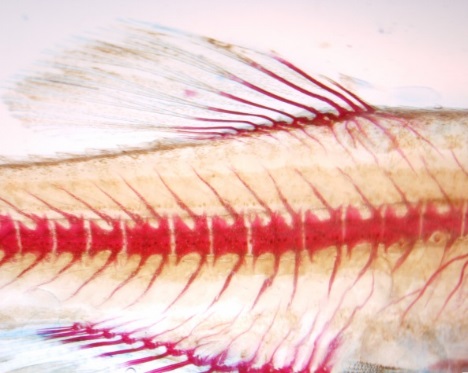


**Supplementary Figure 3. Additional Images of abnormal slc39a8 KO zebrafish after alizarin red staining at 12 weeks post-fertilization.** (A) A single mutant slc39a8 zebrafish demonstrating spinal fusion by fluorescent or light microscopy. (B) Various skeletal abnormalities were observed among slc39a8 KO zebrafish including fused vertebrae and abnormal bone growth along the spine (4 different fish shown). Shown are homozygous mutant slc39a8 zebrafish produced from a cross between two heterozygous mutant slc39a8 zebrafish and genotyped for the mutation. Scale bar 2mm.


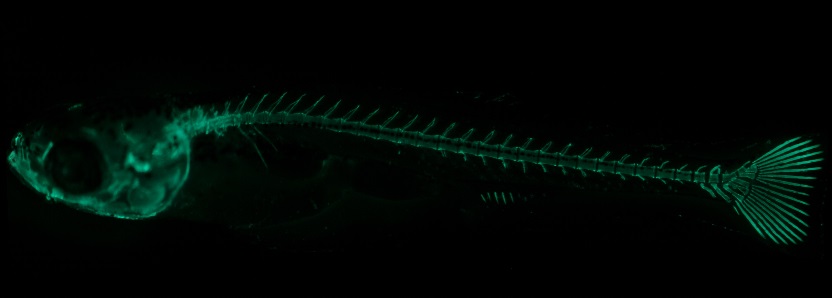

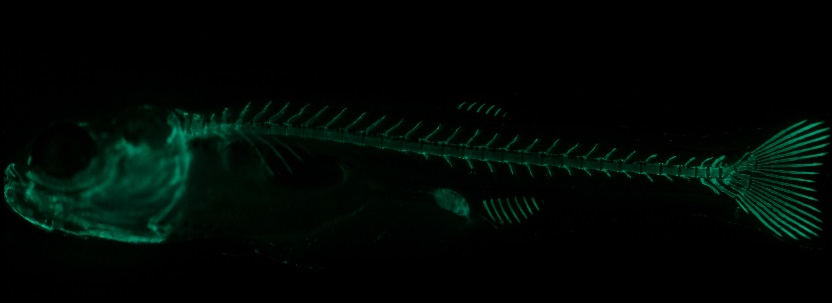

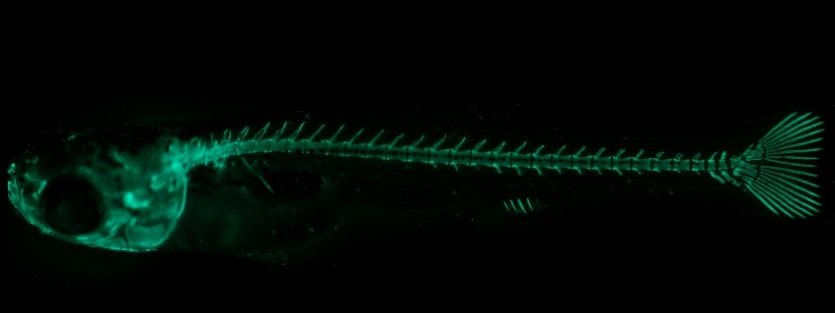


**Supplementary Figure 4. Calcein staining of slc39a8 mutant zebrafish at 13 dpf.** Three example -/- slc39a8 zebrafish are shown. Individual zebrafish were stained with calcein and imaged for fluorescence to look for early skeletal developmental abnormalities. Scale bar 0.5 mm.


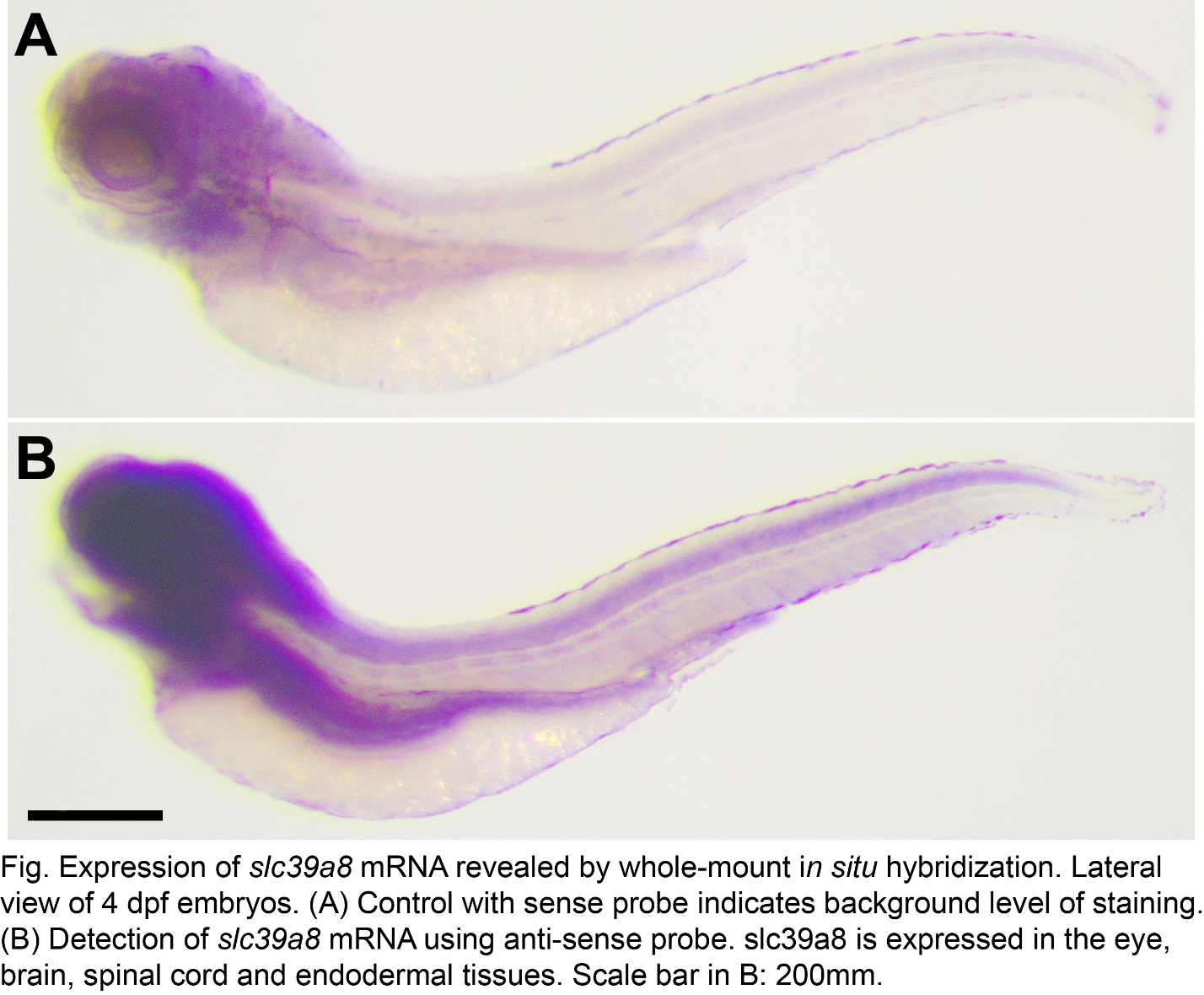


**Supplementary Figure 5. Expression of *slc39a8* mRNA revealed by whole-mount *in situ* hybridization.** Lateral view of 4 days post-fertilization (dpf) zebrafish embryos. (A) Control with sense probe indicating background level of staining. (B) Detection of slc39a8 mRNA using anti-sense probe. Slc39a8 is expressed in the eye, brain, spinal cord and endodermal tissues. Scale bar: 0.2 mm.

**Supplementary Figure 6. Principal components analysis (PCA) of exome sequenced AIS cases, Controls and HapMap reference populations.** EIGENSTRAT was used to calculate PCs for all Exome sequenced individuals and hapmap indivdiuals from CEU, JPT and YRI populations as reference.

**Supplementary Figure 7. Site frequency spectrum of SNPs in the discovery set.** Histogram of the number of SNPs with each listed minor allele frequency in the discovery set. Inlay is a pie chart of the frequencies.

| **Patient DNA Source** | **Case/Control** | **Cohort** | **Number of Samples** | **Genotyping Method** |
| --- | --- | --- | --- | --- |
| Washington University | Cases | Discovery | 267 | Exome |
| Shriners Hospital for Children | Cases | Discovery | 190 | Exome |
| Washington University | Controls | Discovery | 987 | Exome |
|  |  |  |  |  |
| Washington University | Cases | Replication | 288 | KASPAR |
| University of Iowa | Cases | Replication | 146 | KASPAR |
| Hospital for Special Surgery | Cases | Replication | 36 | KASPAR |
| University of Wisconsin | Cases | Replication | 73 | KASPAR |
| Texas Scottish Rite Hospital | Cases | Replication | 272 | KASPAR |
| University of Colorado | Cases | Replication | 42 | KASPAR |
| Washington University | Controls | Replication | 927 | Affy 6.0 |
| Washington University | Controls | Replication | 168 | KASPAR |

**Supplementary Table 1. Patient sample sources for discovery and replication cohorts along with genotype/sequencing platform.** Discovery cohort cases and controls were analyzed identically using the same pipeline. Replication cohorts were genotyped with either the KASPAR genotyping method or Affymetrix 6.0 array. To ensure consistency in calling, 100 individuals in addition to those listed were genotyped on both platforms and found to have 100% concordance.

| **slc39a8 zebrafish in situ probe sequence (833bp)** |
| --- |
| TATCTCGGGCTGGCACTGGGCATCCTGCTGGGCAGTAATTTTGCTCCAAATGCTATCTTTGCCATTGCTGGTGGAATGTTCCTCTACATCTCTCTTGCAGACATGTTCCCAGAGATGAACAGTATCATGGCATCACATACCAAAGACTATCAGGAAAGAGTCGTGTTCTTCCTCATCCAAAATGCTGGACTGCTCACCGGATTCACTATAATTCTGCTTATTACCCTGTTCGCTGGAGATATCAATCTGCAGTAGAAAGAAAGCCAAGGGAAACAAAGAACAATTGTTTTCACTTTCTAAAAAACCAAAAACCCTTCAGACACTATTTTTCTCCCCTATTTTTATATTAAACTTGTGCTGTGTTTGCAGTTGGGAAAATGTAACACAATCAAATGGTCCTGGAAGATTGATTTCTGTAATGTTTTTTTTTTACTGCTGTTGTTGCTCTGCTGTGGCTGTTTGTGTCTGCGAGTTGGCAACAATAGGCCGCTGTGTGTGCAGGAATTCCTCAGTACAGCCCGACACCGAGTCTCTAAGCTGCTTTGTAGAACAAATGTCAAAGCTGCACCTTTTGACTGAGACTGTATATCATGTTTGCAAAGTCCAAGCATAGCACATGAAGTTGAAGACTGTTGATTTGTGCCTTTGTGTCTTTTCTAAATGCTATTATAGGAGGAAGACTGCACTTTATTTAATTACAGTTTAATTTAACATTTAAGTATAAGTAATAATTACATAAAAAAAGGATTAGGTTTGGTTTAGGGTTATTTGTATAGAAATATACATCATTTATTGATTTAGAAAATACAGTGCTTGGCATATATAAGTACACC |

**Supplementary Table 2. Zebrafish *in situ* probe sequence.** Probe was obtained by amplification of cDNA created by RT-PCR of RNA obtained from whole 4 dpf embryos.
