# Supplementary material for: A missense variant in SLC39A8 is associated with severe idiopathic scoliosis
Source: Nat Commun. 2018 Oct 9;9:4171. doi: 10.1038/s41467-018-06705-0 (PMC6177404; doi:10.1038/s41467-018-06705-0)
Supplement: Supplementary file 3 — Description of Additional Supplementary Files [file 41467_2018_6705_MOESM3_ESM.pdf]

Supplementary Dataset 1. Genotype comparisons for 200 AIS patients genotyped at rs13107325 using two methods.
